# Antiparasitic Bromotyrosine Derivatives from the Marine Sponge *Verongula rigida*

**DOI:** 10.3390/md9101902

**Published:** 2011-10-14

**Authors:** Elkin Galeano, Olivier P. Thomas, Sara Robledo, Diana Munoz, Alejandro Martinez

**Affiliations:** 1 Marine Natural Products Research Group, Pharmaceutical Chemistry Faculty, University of Antioquia, Medellin AA 1226, Colombia; E-Mail: amart@farmacia.udea.edu.co; 2 Chemical Institute of Nice, UMR 6001 CNRS, University of Nice—Sophia Antipolis, Parc Valrose, 06108, Nice Cedex 02, France; E-Mail: olivier.Thomas@unice.fr; 3 Program for the Study and Control of Tropical Diseases (PECET), University of Antioquia, Medellin AA 1226, Colombia; E-Mails: sara_robledo@yahoo.com (S.R.); dhyana5@yahoo.com (D.M.)

**Keywords:** bromotyrosines, *Verongula rigida*, antiplasmodial activity, leishmanicidal activity, trypanocidal activity

## Abstract

Nine bromotyrosine-derived compounds were isolated from the Caribbean marine sponge *Verongula rigida*. Two of them, aeroplysinin-1 (**1**) and dihydroxyaerothionin (**2**), are known compounds for this species, and the other seven are unknown compounds for this species, namely: 3,5-dibromo-*N*,*N*,*N*-trimethyltyraminium (**3**), 3,5-dibromo-*N*,*N*,*N*, *O*-tetramethyltyraminium (**4**), purealidin R (**5**), 19-deoxyfistularin 3 (**6**), purealidin B (**7**), 11-hydroxyaerothionin (**8**) and fistularin-3 (**9**). Structural determination of the isolated compounds was performed using one- and two-dimensional NMR, MS and other spectroscopy data. All isolated compounds were screened for their *in vitro* activity against three parasitic protozoa: *Leishmania panamensis*, *Plasmodium falciparum* and *Trypanosoma cruzi*. Compounds **7** and **8** showed selective antiparasitic activity at 10 and 5 μM against *Leishmania* and *Plasmodium* parasites, respectively. Cytotoxicity of these compounds on a human promonocytic cell line was also assessed.

## 1. Introduction

Tropical diseases caused by single-celled parasites, like malaria, leishmaniasis and Chagas disease, are of particular importance in tropical regions of the world. They represent the three most important diseases caused by parasitic protozoa. It is estimated that these diseases are responsible for more than 900,000 deaths every year [1–3]. In the absence of a long-term protective vaccine, the control of these parasitic infections is based on a few chemotherapeutic agents. Most of these agents are now facing parasitic resistance, severe adverse effects and variable efficiency according to the phase of the disease. For these reasons, the search for new, safe, and effective antiprotozoal agents is urgent [4].

In this context, we evaluated the potential of Colombian sponges as sources of antiparasitic compounds. Urabá Gulf is located in the Southwestern Caribbean Sea, on the border with Panama. The sponge biodiversity of this Colombian region has been poorly studied so far. We have already investigated the antimicrobial, antiparasitic and antitumoral activity of the extracts of some sponges of this area, and *Verongula rigida* (Esper 1794, Verongida, Aplysinidae) appeared of high interest for its chemical composition [5–7]. This species, like other Verongida marine sponges, are of much biological and chemical interest. This group of sponges is known to produce brominated metabolites that are biogenetically derived from tyrosine [8]. For this reason, bromotyrosine metabolites have been considered as potential chemotaxonomic markers of Verongida sponges [9,10]. A wide range of biological activities has been reported for some of these secondary metabolites, including antimicrobial, anti-enzymatic, cytotoxic and antiparasitic activities [11–13]. Previous studies on the sponge *V. rigida* led to the discovery of antimicrobial and enzymatic activity of its extracts [14,15] and the isolation and structure identification of bromotyrosine-derived compounds [8,16,17].

In the present work, nine isolated compounds were evaluated against the most important tropical parasitic diseases: malaria, leishmania and Chagas. The selectivity indices were measured by dividing the antiparasitic activity of the compounds by their cytotoxicity against the promonocytic macrophage cell line U937.

## 2. Results and Discussion

**Figure 1:**
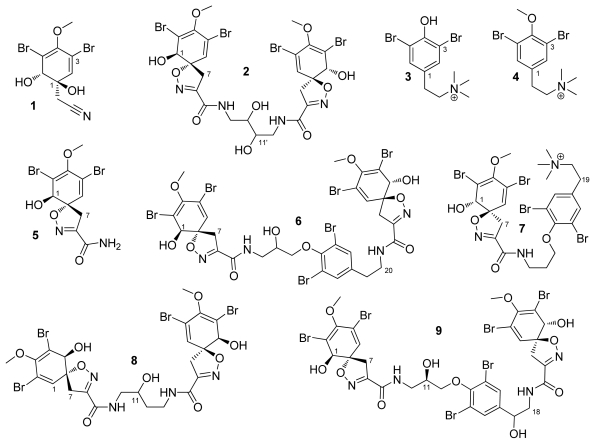
Bromotyrosine-derivatives isolated from the marine sponge *Verongula rigida*.

Chemical purification of a methanol-dichloromethane (1:1, v/v) extract of *V. rigida* afforded nine compounds (Figure 1), two of them known compounds for the species: aeroplysinin-1 (**1**), which was first isolated from *Ianthella ardis* (Laubenfels, 1950), is known today as *Aiolochroia crassa* (Hyatt, 1875) in 1970 [18]. This compound shows antimicrobial and cytotoxic activities and also inhibits the growth of endothelial cells in culture in the micromolar range (antiangiogenic activity) [19,20]. Dihydroxyaerothionin (**2**) was first isolated from *V. rigida* in 1989 [17], but no biological activity has been reported so far.

Seven unknown compounds for this species, but known in other species, were isolated. 3,5-dibromo-*N*,*N*,*N*-trimethyltyraminium (**3**) was reported from *Aplysina fistularis* (Pallas, 1766) as a dual adrenergic agent [21]. 3,5-dibromo-*N*,*N*,*N*,*O*-tetramethyltyraminium (**4**) was isolated from *Verongula* sp. in 1994 [22], without any reference to biological activity. Purealidin R (**5**) was first reported from *Psammaplysilla purpurea* (Carter, 1880); known as *Pseudoceratina purpurea* (Carter, 1880) [23], without any bibliography report of biological activity. 19-deoxyfistularin 3 (**6**) was isolated from the sponge *Verongia* sp. [24] without any report of biological activity. Purealidin B (**7**) was isolated from *Psammaplysilla purpurea* and showed no cytotoxicity, but it exhibited antimicrobial activity against *Candida albicans*, *Cryptococcus neoformans*, *Paecilomyces variotii*, *Staphylococcus aureus*, *Sarcina lutea* and *Bacillus subtilis* [25]. This molecule also has been isolated from the sponges *Pseudoceratina verrucosa* and *Pseudoceratina crassa* [26]. 11-hydroxyaerothionin (**8**) was isolated from the sponge *Pseudoceratina durissima* (Carter, 1885) and it showed antimicrobial activity against *Staphylococcus aureus*, *Bacillus subtilis*, *Candida albicans* [27] and anti-tuberculosis activity against *Mycobacterium tuberculosis* with wake cytotoxicity reported [28]. Other evaluated activities were cytotoxicity on human tumor cells [29] and as an adenosine A1 receptor inhibitor [30]. Fistularin-3 (**9**) was isolated in 1979 from the sponge *Aplysina fulva* (Pallas, 1766) [31]. It has been evaluated against *Mycobacterium tuberculosis* H37Rv, cytotoxicity activity against J744 macrophages [32], human breast carcinoma cell line MCF-7 activity [33] and feline leukemia virus activity [34]. The structures were determined by NMR (1D and 2D), MS data analysis and literature comparisons.

All compounds were assayed using the same biological activity protocol. Antimalarial, leishmanicidal, anti-chagas disease and cytotoxic activities were analyzed in triplicate (Table 1). Compounds with high cytotoxicity and weak activity over axenic amastigotes of *Leishmania panamensis* were not analyzed over intracellular amastigotes of *Leishmania*, instead they were considered as compounds without potential leishmanicidal activity due to their low selectivity. Compound **8** showed 12.6% inhibition of intracellular amastigotes of *Leishmania* at 10 μM and it did not exhibit cytotoxicity at 20 μM. A similar case of selectivity occurs with compound **7**. It showed 23.2% inhibition *in vitro* over *P. falciparum* at 5 μM, and it did not exhibit cytotoxic activity at 20 μM. Currently, these two molecules are underdoing further studies on this biological selectivity. Compound **1** showed 29.1% of parasite growth inhibition *in vitro* over *T. cruzi* at 10 μM, but it exhibits a high cytotoxicity (94.8%) at 20 μM. These high bioactivities can be explained by the presence of a very reactive cyanide group in its structure, which has been reported to be an inhibitor of the enzyme cytochrome C oxidase, preventing transport of electrons to produce ATP, causing cell apoptosis [35]. Compounds **2** and **4** did not exhibit antiprotozoal activity *in vitro* and they have moderate cytotoxicity at 20 μM. Compounds **5** and **9** are weak antiparasitic compounds (inhibiting less than 11% at 10 μM) and they produce the 45.3% and 58.2% growth inhibition over U-937 cells at 20 μM, showing weak selectivity. Compound **1** is considered to be the most cytotoxic agent evaluated. Compounds **3** and **6** showed no cytotoxic or antiparasitic activity. In general, *Plasmodium* parasite was more sensitive to bromotyrosines compounds than the *Leishmania* and *Trypanosoma* parasites evaluated.

## 3. Experimental Section

### 3.1. General Experimental Procedures

Optical rotations were measured on a BTI-162 polarimeter, while UV measurements were performed on a Varian Cary 300 Scan UV–visible spectrophotometer. Infrared spectra were acquired on a PerkinElmer Paragon 1000 FT-IR spectrophotometer. NMR data were collected on a Bruker Avance 500 MHz spectrometer using deuterated NMR solvents supplied by Sigma-Aldrich. Spectra were referenced to residual ^1^H and ^13^C in the deuterated solvents. Low resolution electrospray ionisation (ESI) mass spectra were obtained with a Bruker Esquire 3000 Plus spectrometer in the positive or negative mode by direct injection method. The solvents used (MeOH, MeCN and H_2_O) were HPLC grade and obtained from Merck. Trifluoroacetic acid (TFA) used was HPLC grade and supplied by Sigma-Aldrich. HPLC purifications were carried out on a Waters 600 system equipped with a Waters 717 plus autosampler, a Waters 996 photodiode array detector and a Sedex 55 evaporative light scattering detector (Sedere, France).

### 3.2. Sponge Material

A specimen of the marine sponge *V. rigida* was collected at a depth of about 10 m from Urabá Gulf, Caribbean Sea, Colombia (8°40′14″N, 77°21′28″W) in October 2008 and identified by Sandra Ospina. A voucher sample (INV-POR 0065) has been deposited in the sponge collection of Museo de Historia Natural Marina de Colombia, Invemar. The sponge was kept frozen at −20 °C from collection until the extraction process.

### 3.3. Extraction and Isolation

A portion of *V. rigida* (280 g wet) was freeze-dried and ground to obtain a dry powder (50 g), which was extracted three times with a mixture of MeOH/CH_2_Cl_2_ (1:1) at room temperature for 15 min in an ultrasonic bath to give 15.9 g of a crude extract after concentration under reduced pressure. The crude extract was fractionated by RP-C18 vacuum liquid chromatography (elution with 500 mL of each solvent in a decreasing polarity gradient of H_2_O 100% (F1, 8.7 g), H_2_O–MeOH 1:1 (F2, 1.1 g), H_2_O–MeOH 1:3 (F3, 0.6 g), MeOH 100% (F4, 1.2 g), MeOH–CH_2_Cl_2_ 3:1 (F5, 0.8 g) and CH_2_Cl_2_ 100% (F6, 0.08 g)). Samples were further purified by phenyl-hexyl semi-preparative HPLC column chromatography (Phenomenex Gemini, 10 mm × 250 mm, 5 μm, 3.0 mL/min) using gradient elution from 20% MeCN + 0.1% TFA to 100% over 30 min. From F2 were isolated: **1** (2.6 mg, 2.2% w/w), **2** (0.8 mg, 0.7%), **3** (3.4 mg, 3.3%), **4** (3.8 mg, 2.9%), **5** (1.9 mg, 1.6%), **6** (1.4 mg, 1.2%), **7** (1.7 mg, 1.4%), **8** (1.1 mg, 0.9%) and **9** (4.2 mg, 3.5%).

Aeroplysinin-1 (**1**): Yellow solid; ESI-MS *m/z* 335.6 (49%), 337.6 (100%), 349.6 (51%), C_9_H_9_Br_2_NO_3_ 338.98. Spectroscopic data matched those previously published [36].

Dihydroxyaerothionin (**2**): Light yellow solid; ESI-MS *m/z* 868.9 (9%), 870.9 (38%), 873.0 (76%), 875.0 (28%), 876.8 (8%), C_24_H_26_Br_4_N_4_O_10_Na^+^ 873.10. Spectroscopic data matched those previously published [17].

3,5-dibromo-*N*,*N*,*N*-trimethyltyraminium (**3**): Light brown solid; UV (MeOH) λ_max_ (log ɛ) 220.5 (4.30), 288.5 (3.00); IR (neat) 3422, 2955, 1630 (arom), 1543, 1425, 1262, 1032 and 650 cm^−1; 1^H NMR data (500 MHz, DMSO-*d*_6_) δ 7.51 s (2H, H-2, H-6), 6.81 s (OH), 2.94 t (2H, H-7), 3.46 m (2H, H-8), 3.09 s (9H, N-Me3); ^13^C and (125 MHz, DMSO-*d*_6_) δ 120.5 (C-1, s), 132.6 (C-2, C-6, d), 112.1 (C-3, C-5, s), 142.7 (C-4, s), 26.7 (C-7, t), 65.63 (C-8, t) and 52.32 (N-Me3); ESI-MS *m/z* 336.0 (48%), 338.0 (100%), 340.0 (50%), 341.0 (5%), C_11_H_16_Br_2_NO^+^ 338.06. Spectroscopic data matched those previously published [37].

3,5-dibromo-*N*,*N*,*N*,*O*-tetramethyltyraminium (**4**): Brown solid; UV (MeOH) λ_max_ (log ɛ) 218.2 (4.21), 277.1 (3.18), 282.2 (3.16); IR (neat) 2570, 1635 (arom), 1440 and 622 cm^−1; 1^H NMR data (500 MHz, DMSO-*d*_6_) δ 7.69 s (2H, H-2, H-6), 3.03 m (2H, H-7, *J =* 5.2, 12.1, 17.2 Hz), 3.50 m (2H, H-8, *J =* 4.9, 12.0, 17.3 Hz), 3.78 s (3H, OMe), 3.10 s (9H, N-Me3); ^13^C and (125 MHz, DMSO-*d*_6_) δ 133.4 (C-1, s), 135.7 (C-2, d), 117.4 (C-3, C-5, s), 152.3 (C-4, s), 135.9 (C-6, d), 26.9 (C-7, t), 65.3 (C-8, t), 60.4 (OMe), 52.42 (N-Me), 52.39 (N-Me), 52.36 (N-Me); ESI-MS *m/z* 350.1 (61%), 352.0 (100%), 354.0 (56%), C_12_H_18_Br_2_NO^+^ 352.08. Spectroscopic data matched those previously published [22].

Purealidin R (**5**): Yellow solid; UV (MeOH) λ_max_ (log ɛ) 283.4 (3.25); IR (neat) 3440, 2960, 2260, 1655, 1150, 1120 and 710 cm^−1; 1^H NMR data (500 MHz, DMSO-*d*_6_) δ 7.84 br s (1H, N-H), 7.59 bs s (1H, N-H), 6.57 s (1H, H-5), 6.36 d (1H, C1-OH, *J =* 8.2), 3.91 d (1H, H-1, *J =* 7.7), 3.01 d (1H, H-7a, *J =* 17.4), 2.89 d (1H, H-7b, *J =* 17.4), 3.65 s (3H, OMe); ^13^C and (125 MHz, DMSO-*d*_6_) δ 74.9 (C-1, s), 114.2 (C-2, s), 153.2 (C-3, s), 116.5 (C-4, s), 134.5 (C-5, d), 90.2 (C-6, s), 41.1 (C-7, t), 159.6 (C-8, s), 162.6 (C-9, s), 59.81 (OMe); ESI-MS *m/z* 380.7 (50%), 382.7 (100%), 384.7 (8%), C_10_H_10_Br_2_N_2_O_4_ 382.01. Spectroscopic data matched those previously published [23].

19-deoxyfistularin 3 (**6**): Red-brown solid; UV (MeOH) λ_max_ (log ɛ) 228.4 (4.10) and 284.2 (3.78); IR (neat) 3440, 1655, 1610, 1535, 1420, 1040 and 720 cm^−1; 1^H NMR data (500 MHz, DMSO-*d*_6_) δ 7.72 s (2H, H-15, H-17), 6.52 s (1H, H-5), 6.53 s (1H, H-5′), 4.26 m (1H, H-11), 4.18 s (1H, H-1), 4.19 s (1H, H1′), 4.07 m (2H, H-12), 2.88 (2H, H-19, und. solvent.), 3.84 (2H, H-7b, H-7b′, *J =* 18.3), 3.76 m (1H, H-10a), 3.72 s (6H, OMe), 3.56 td (2H, H-20, *J =* 6.9, 3.4), 3.52 m (1H, H-10b), 3.18 (2H, H-7a, H-7a′, *J =*18.3); ESI-MS *m/z* 1091.6 (0.9%), 1092.7 (15%), 1094.6 (67%), 1095.6 (16%), 1096.7 (100%), 1097.7 (23%), 1098.6 (75%), 1099.5 (18%), 1100.6 (21%), C_31_H_30_Br_6_N_4_O_10_ 1098.01. Spectroscopic data matched those previously published [24].

Purealidin B (**7**): Colorless solid; UV (MeOH) λ_max_ (log ɛ) 219.8 (3.69) and 283.4 (2.69); IR (neat) 3440, 2975, 2870, 1680, 1465, 1360, 1200, 1150 and 680 cm^−1; 1^H NMR data (500 MHz, Acetone-*d*_6_) δ 7.68 s (2H, H-15, H-17), 6.53 s (1H, H-5), 4.22 s (1H, H-1), 4.10 t (2H, H-12), 3.84 d (1H, H-7a, *J =* 18.1), 3.83 s (3H, OMe), 3.73 s (9H, N-Me3), 3.61 t (2H, H-10 *J =* 6.91), 3.56 m (2H, H-20), 3.22 d (1H, H-7b *J =* 18.2), 3.18 m (2H, H-19), 2.02 m (2H, H-11); ESI-MS *m/z* 758.0 (11%), 760.0 (54%), 762.0 (68%), 764.0 (46%), 765.9 (10%), C_24_H_30_Br_4_N_3_O_5_ ^+^ 760.13. Spectroscopic data matched those previously published [25].

11-hydroxyaerothionin (**8**): Colorless solid; UV (MeOH) λ_max_ (log ɛ) 227.6 (3.97) and 282.2 (3.74); IR (neat) 3440, 3330, 1650, 1200, 1150 and 690 cm^−1; 1^H NMR data (500 MHz, Acetone-*d*_6_) δ 7.79 br t (1H, N-H *J =* 5.2), 7.55 br t (1H, N-H *J =* 5.4), 6.54 s (2H, H-5, H-5′), 5.44 br s (1H, OH), 4.20 s (1H, H-1′), 4.19 s (1H, H-1), 3.86 d (1H, H-7a, *J =* 18.3), 3.85 d (1H, H-7′a, *J =* 18.0), 3.85 m (1H, H-11), 3.74 s (6H, OMe), 3.55 m (2H, H-13a), 3.47 m (1H, H-10a), 3.42 m (1H, H-13b), 3.30 dd (1H, H-10b, *J =*13.5), 3.19 d (1H, H-7b, *J =* 18.3), 3.20 d (1H, H-7′b, *J =* 18.3), 1.79 m (1H, H-12a), 1.63 m (1H, H-12b); ESI-MS *m/z* 852.0 (14%), 854.0 (72%), 856.0 (100%), 858.0 (55%), 860.2 (8%), C_24_H_26_Br_4_N_4_O_9_Na^+^ 783,13. Spectroscopic data matched those previously published [27].

Fistularin-3 (**9**): Colorless solid; UV (MeOH) λ_max_ (log ɛ) 220.6 (4.17) and 284.6 (3.77); IR (neat) 3640, 3350, 1650, 1520 and 690 cm^−1; 1^H NMR data (500 MHz, Acetone-*d*_6_) δ 7.66 s (2H, H-15, H-17), 6.53 s (1H, H-5), 6.52 s (1H, H-5′), 4.90 dd (1H, H-19 *J =* 4.5, 7.2), 4.24 td (1H, H-11 *J =* 5.6, 10.2, 10.2), 4.19 s (1H, H-1), 4.17 s (1H, H-1′), 4.05 ddd (2H, H-12 *J =* 6.0, 9.3, 19.3), 3.82 d (1H, H-7b, *J =* 18.2), 3.81 d (1H, H-7′b *J =* 18.2), 3.78 dd (1H, H-10a *J =* 4.5, 13.7), 3.73 s (6H, OMe), 3.61 m (1H, H-20a), 3.56 m (1H, H-10b), 3.50 m (1H, H-20b), 3.20 d (1H, H-7a, *J =* 18.1), 3.16 d (1H, H-7′a, *J =* 17.5); ESI-MS *m/z* 1131.1 (1%), 1132.2 (2%), 1133.1 (1%), 1134.3 (8%), 1135.4 (2%), 1136.3 (4%), 1138.2 (5%), 1139.5 (2%), 1140.2 (2%), C_31_H_30_Br_6_N_4_O_11_Na^+^ 1137.01. Spectroscopic data matched those previously published [38].

### 3.4. Bioassays

#### 3.4.1. *In Vitro* Leishmanicidal Activity on Axenic and Intracellular Amastigotes

Axenic and intracellular amastigotes of GFP-transfected *L.* (V.) *panamensis* strain (MHOM/CO/87/UA140epir GFP) were used for the *in vitro* testing of leishmanicidal activity.

##### 3.4.1.1. Activity against Axenic Amastigotes

The ability of compounds to kill axenic amastigotes of *L.* (V.) *panamensis* was determined based on the viability of the parasites evaluated by the MTT (3-(4,5-dimethylthiazol-2-yl)-2,5-diphenyltetrazolium bromide) method as previously described [39]. In brief, parasites were cultivated in Schneider’s medium at pH 5.4 supplemented with 20% heat-inactivated FBS (incubated for 3 days at 32 °C). Afterwards they were harvested, washed and resuspended at 2 × 10^6^ axenic amastigotes/mL in fresh medium. Each well of a 96-well plate was seeded with 100 μL of each parasite suspension and 100 μL of the test compound at 20 μM as final concentration, was evaluated. Plates were incubated at 32 °C. After 72 h of incubation the effect of the drugs was determined by adding 10 μL/well of MTT and incubating at 32 °C for 3 h. The reaction was stopped and the quantity of formazan produced was measured with a Bio-Rad ELISA reader set at 570 nm. Parasites cultivated in the absence of the compound but maintained under the same conditions were used as controls for growth and viability. Parasites cultivated in the presence of anphotericin B were used as positive controls for leishmanicidal activity.

##### 3.4.1.2. Activity against Intracellular Amastigotes

The effect of the compounds against intracellular amastigotes of *L.* (V.) *panamensis* was evaluated by flow cytometry. Briefly, U937 cells were dispensed in 24-well plates at a concentration of 300,000 cells/well, which were treated with 1 μM of phorbol myristate acetate (PMA) for 48 h at 37 °C, after which they were infected with promastigotes of *L.* (V.) *panamensis* in stationary growth phase (day 5) in modified NNN medium, at a 1:25 cell/parasite. After 3 h of incubation at 34 °C in 5% CO_2_, non-internalized parasites were washed and incubated again at 34 °C and 5% CO_2_ to allow differentiation to amastigote’s form. After 24 h of incubation, the compound at 10 μM was added. Infected and treated cells were maintained at 34 °C and 5% CO_2_ for 72 h. The leishmanicidal effect was measured in a flow cytometer at 488 nm of excitation and 525 nm of emission [40]. Infected cells exposed to amphotericin B were used as a positive control for leishmanicidal activity.

#### 3.4.2. Antimalarial Activity against Plasmodium Falciparum

Antimalarial activity was evaluated against *P. falciparum* NF54 strain in asynchronous cultures. The assay was carried out with *P. falciparum* in 24-well suspension cultures using O positive human serum, 2% hematocrit in RPMI-1640 medium supplemented with Hepes, hypoxanthine, glutamine, dextrose and the test compound at 5 μM/well. Cultures were maintained at 37 °C for 48 h under a 1% O_2_, 4% CO_2_, and 95% N_2_ atmosphere. Chloroquine was used as a positive activity control. Antiplasmodial activity was determined by DNA analysis using a fluorometric method with ethidium bromide dye (EtBr), and fluorescence was read at emission 510 nm and excitation 590 nm [41].

#### 3.4.3. Trypanocidal Activity

The *in vitro* antitrypanosomal activity was evaluated against *T. cruzi* Tulahuen strain. U937 cells in wells of a 96-well plate containing RPMI medium were infected with stationary-phase epimastigotes at a 5:1 parasite:cell proportion. After 24 h, the test compound was added at 10 μM. Beznidazol was used as a positive control. The effect was analyzed colorimetrically for β-galactosidase activity [42] 72 h later in a spectrophotometer at 570 nm.

#### 3.4.4. *In Vitro* Cytotoxic Activity in Mammalian Cells

Cytotoxic activity of compounds was assessed based on the viability of the human promonocytic cell line U937 (ATCC CRL-1593.2™) evaluated by the MTT (3-(4,5-dimethylthiazol-2-yl)-2, 5-diphenyltetrazolium bromide) method [41]. Briefly, cells were grown in 96-wells plates at 100,000 cells/mL in RPMI-1640 supplemented with 10% FBS and the compound at 20 μM in duplicate. The cells were incubated at 37 °C with 5% CO_2_ in air for 72 h in the presence of the compounds, and then the effect of the drug was determined using an MTT assay as described above by adding 10 μL/well of MTT solution (0.5 mg/mL) and incubating at 37 °C for 3 h. The reaction was stopped by adding a 50% isopropanol solution with 10% sodium dodecyl sulfate for 30 min. Cell viability was determined based on the quantity of formazan produced, which was measured with a Bio-Rad ELISA reader set at 570 nm. As a viability test, cultured cells in the absence of extracts were used. Amphotericin B was used as a cytotoxicity control.

## 4. Conclusions

This is the first report regarding seven of the nine bromotyrosine-derivatives from the sponge *V. rigida* (**3**–**9**) and the first biological activity reports for compounds **2**, **4**, **5** and **6**. None of the isolated compounds had been previously evaluated against malaria, Leishmania and Chagas disease, and for this reason, this work is the first report to consider these bromotyrosines as potential antiparasitic agents. The results demonstrate that some of the compounds, such as compounds **7** and **8**, are interesting *in vitro* against *Plasmodium* and *Leishmania* parasites, respectively.

Compound **7** is structurally close to psammaplysin-H, isolated from the sponge *Pseudoceratina* sp., which was found to display a potent and selective activity against *Plasmodium falciparum*. In this work we also noticed a high selective bioactivity against axenic *Leishmania parasites*. In the same manner, compound **8** displayed a high selective index against both *Plasmodium* and *Leishmania* parasites. Previous reports of compounds **8** as an anti-tuberculosis agent, have suggested that hydroxylation at position 11 is essential for the activity of this compound. In the compound **2**, a dimer of compound **8**, there are two hydroxyl groups at positions 11 and 11′. Since this compound is less bioactive than compound **8**, it is likely that the double hydroxylation in the compound **2** forms a steric hindrance.

Compounds with hydroxylation at positions 11 and the presence of a 2,6-dibromophenyl radical linking two units of spirocyclohexadienylisoxazolines, like compounds **6** and **9**, show reductions in their antiparasitary activities compared with the molecules with hydroxylation at positions 11 and without the 2,6-dibromophenyl radical. Compounds **5** and **9** showed no cytotoxic or antiparasitic activity, and this proves that the existence of halogen atoms in molecules is not an indicator of bioactivity and/or cytotoxicity. Compounds **7** and **8** are interesting reference points for the development of new related antiparasitic substances. They are currently being evaluated to determine a higher selectivity dosage and further investigations may include the assessment of their *in vivo* efficacy in animal models, which could not be performed in the current study due to the limited amount of compounds available.

## Figures and Tables

**Table 1:** *In vitro* antiparasitic and cytotoxic activities of sponge-isolated compounds **1**–**9**.

Compound	% of inhibition of the growth a				

	U-937 cells (20 μM)	*L. panamensis*		*P. falciparum* Total forms (5 μM)	*T. cruzi* Intracellular amastigotes (10 μM)

		Axenic amastigotes (20 μM)	Intracellular amastigotes (10 μM)		
**1**	94.8 ± 3.6	0	NE	35.3 ± 3.5	29.1 ± 0.4
**2**	8.2 ± 1.7	0.3 ± 0.06	2.1 ± 0.4	7.9 ± 1.2	0
**3**	0	0	NE	0	0
**4**	5.3 ± 1.1	0	NE	0	0
**5**	45.3 ± 13.5	0	NE	7.1 ± 1.2	1.6 ± 0.3
**6**	0	0	NE	0	0.2 ± 0.03
**7**	0	1.6 ± 0.4	0	23.2 ± 1.0	0
**8**	0	0.0	12.6 ± 0.9	8.0 ± 0.5	0
**9**	58.2 ± 12.0	7.7 ± 1.6	NE	10.8 ± 1.5	6.3 ± 1.3
Amphotericin B b	53.2	60.4 ± 5.7	44.9 ± 7.1	NA	NA
Chloroquine c	NA	NA	NA	66.8 ± 1.3	NA
Benznidazole d	NA	NA	NA	NA	44.5 ± 2.7

a Percentage of inhibition corresponds to the inhibition of the U-937 cells or parasites growth determined by colorimetric MTT method (for U-937 cells and axenic amastigotes of *L. panamensis*), flow cytometry (for intracellular amastigotes of *L. panamensis*), fluorometry (for *P. falciparum* total forms) and colorimetric β-galactosidase method (for *T. cruzi* intracellular amastigotes). Data are expressed as the average from at least two independent experiments, each done in triplicate;

b Lethal Concentration 50 (LC_50_) for U-937 cells (previously determined in our lab) = 33.2 μM; Effective Concentration 50 (EC_50_) for axenic and intracellular amastigotes of *L. panamensis* (previously determined in our lab) = 0.05 μM and 0.04 μM, respectively;

c EC_50_ for total forms of *P. falciparum* (previously determined in our lab) = 42.6 μM;

d EC_50_ for intracellular amastigotes of *T. cruzi* (previously determined in our lab) = 9.3 μM. NE: Not evaluated due to the high toxicity level; NA: Not applicable because these drugs are not used for these parasites.
